# HAE-Net: A hierarchy-aware enhanced method for 3D panoptic segmentation in orchard scenes

**DOI:** 10.1016/j.plaphe.2026.100273

**Published:** 2026-09-10

**Authors:** Shuo Zhang, Yiqun Wang, Lianchuan Shi, Ze Ji, Xiude Chen, Weikuan Jia

**Affiliations:** a School of Computer Science and Artificial Intelligence, Shandong Normal University, Jinan, 250358, China; b Shandong Key Laboratory of Intelligent Collaborative Perception and Computing, Jinan, 250358, China; c School of Engineering, Cardiff University, Cardiff, CF24 3AA, United Kingdom; d College of Mechanical and Electrical Engineering, Hohai University, Changzhou, 213200, China; e National Research Center for Apple Engineering and Technology, Shandong Agricultural University, Taian, 271018, China

**Keywords:** 3D point cloud segmentation, Panoptic segmentation, Hierarchical segmentation, Attention mechanism, Precision agriculture

## Abstract

Reliable perception of orchard scenes is needed for agricultural automation. Fruit detection, counting, and localization support yield prediction, harvest planning, and precision fertilization, while 3D point clouds provide geometric information with less dependence on ambient illumination than conventional RGB images. Orchard analysis also requires predictions at more than one scale: individual fruits and trunks must be separated, but the fruits must still be associated with their parent tree. Most existing 3D panoptic segmentation methods operate at a single instance granularity and do not represent this hierarchy. We propose HAE-Net, a hierarchy-aware segmentation method built on a shared encoder and multiple decoders. At the encoder output, the Hierarchical Feature Enhancement (HFE) module supplies global aggregation for tree-level grouping, multi-scale context for semantic classification, and local boundary information for standard-instance separation. The Cross-Decoder Attention Gate (CDAG) regulates the encoder features transferred at each skip connection. Its spatial, channel, multi-scale, and pixel-level components allow the three decoders to use different mixtures of the shared representation. A Hierarchical Consistency Loss (HCL) links the two instance granularities by constraining the predicted centers of component instances and their parent tree instance. We evaluate HAE-Net on HOPS (Hierarchical Orchard Panoptic Segmentation), which includes point clouds acquired with terrestrial laser scanners, uncrewed aerial vehicles, uncrewed ground vehicles, and SfM reconstruction. Quantitative comparisons and ablations use the labeled TLS validation split. HAE-Net achieves an mIoU of 71.63% and an mPQ of 53.96%, exceeding HAPT3D by 9.81 and 5.43 percentage points, respectively. Because ground-truth labels for the four test subsets are not publicly accessible, their predictions are used only for qualitative inspection.

## 1 Introduction

Agriculture is a foundational sector of the economy, and crop-yield estimation is a core task in precision agriculture [1,2]. Accurate and timely yield estimates support harvest planning, production forecasting, and agricultural resource allocation [2]. In orchard management, accurate fruit detection, counting, and localization are prerequisites for yield estimation and automated harvesting and can help alleviate agricultural labor shortages and improve production efficiency [[3], [4], [5]].

Traditional yield-estimation methods rely primarily on manual sampling or 2D image analysis. Manual sampling is labor-intensive and subject to observer variability, making it difficult to meet the management needs of large orchards. Although 2D image-based methods provide a degree of automation, their single-view imaging process remains sensitive to fruit occlusion, illumination variation, and complex backgrounds [6]. With the development of 3D sensing technologies such as LiDAR, RGB-D cameras, and Structure from Motion (SfM), point-cloud-based scene understanding has become increasingly applicable to agricultural environments [7]. Three-dimensional point clouds directly represent scene geometry and depth, providing information that is unavailable from a single 2D view. Combining 2D instance segmentation with SfM-derived geometry increased apple-detection F1 from 0.816 to 0.881, although the SfM stage was too slow for real-time operation [6]. However, the volume of 3D data entails an accuracy–efficiency trade-off: the HOPS dataset divides aggregated orchard-row point clouds into tiles containing one million points on average [8]. When coverage and measurement quality are adequate, retaining high-resolution observations can preserve geometric information that may be lost during quantization and provide richer local cues for feature extraction [9]. Such observations can also supply more candidate correspondences for registration; in multi-view reconstruction of obscured tree branches, registration increased the number of corresponding points by at least 24% [10]. However, larger point sets increase storage and data-transfer requirements and raise the computational and GPU-memory costs of neighborhood search, feature extraction, training, and inference [11,12]. Richer point clouds may also contain more noise, whereas random point sampling or coordinate quantization can discard key features and fine geometric details, which may impair fine-grained segmentation [[9], [10], [11]]. This trade-off motivates the use of sparse convolutions for large-scale point-cloud processing. MinkowskiEngine [13] provides an efficient sparse-convolution implementation that applies standard convolution operations to voxelized point clouds. Building on this implementation, MinkUNet [13] extends the U-Net architecture to 3D sparse convolutions and produces point-wise predictions through an encoder–decoder structure with skip connections.

Annotated agricultural point clouds now cover several crops and acquisition settings. BUP20 [14] provides RGB-D data of greenhouse sweet peppers for instance-segmentation research, and Crops3D [15] contains organ-level annotations for multiple crop varieties. Fuji-SfM [6,16] supplies SfM-reconstructed point clouds of apple trees. The HOPS (Hierarchical Orchard Panoptic Segmentation) dataset [8] focuses on hierarchical panoptic segmentation in orchards and combines terrestrial laser scanning (TLS), uncrewed aerial vehicle (UAV), uncrewed ground vehicle (UGV), and SfM data. Its labeled splits include semantic, standard-instance, and tree-instance annotations.

Panoptic segmentation provides both point-wise semantic labels and instance-level object identities within a unified scene representation [17]. Compared with independent semantic or instance segmentation, it answers both “what is it?” and “which instance is it?” Panoptic segmentation has progressed in autonomous driving [18], indoor scene understanding [19], and robotic navigation. In the 3D point-cloud domain, methods generally follow bottom-up or top-down strategies. Bottom-up methods predict semantic labels and instance embeddings for each point and then group points with similar embeddings using clustering algorithms such as HDBSCAN [20] or Mean Shift [21]. Panoster [22] predicts point-wise offsets toward instance centers, while EfficientLPS [23] uses efficient feature aggregation. Top-down methods first detect objects and then segment the points inside their bounding boxes. For example, 3D-BoNet [24] regresses 3D bounding boxes and predicts point-wise masks. Transformer-based methods such as Mask3D [25] extend query-based mask prediction to 3D point clouds.

Recent work has broadened 3D panoptic segmentation from point-cloud-only pipelines toward multimodal and prior-guided perception. IAL addresses image–LiDAR alignment through modality-synchronized augmentation and a transformer decoder, reducing sparsity-related errors in LiDAR panoptic segmentation [26]. Panoptic-VSNet further incorporates visual–semantic priors, instance-aware superpixel fusion, and adaptive panoptic prediction for multimodal 3D panoptic segmentation [27]. In agricultural point-cloud analysis, recent studies have also moved from semantic segmentation toward organ- and plant-level instance segmentation for phenotyping [[28], [29], [30], [31]]. These advances show the growing importance of structured 3D scene understanding, but their instance definitions differ from the row-scale orchard setting considered here. Most of them operate at one instance granularity, target single plants, or focus on urban-scene objects, whereas orchard yield attribution requires each fine-grained fruit or trunk instance to remain linked to its coarse parent tree instance.

Multi-task learning seeks to improve related predictions through shared representations [32]. In scene understanding, a common multi-scale architecture has been applied separately to depth prediction, surface-normal estimation, and semantic labeling [33], while task-specific edge prediction has been incorporated into an end-to-end semantic-segmentation system to improve boundary localization [34]. In agriculture, joint objectives have included classifying aphid presence and estimating aphid size [35], as well as detecting weeds and segmenting drivable areas [36]. Balancing heterogeneous losses remains difficult in these systems. Fixed or manually tuned weights do not change during training; uncertainty-based weighting instead learns task-dependent weights [37], whereas GradNorm adjusts gradient magnitudes to balance training rates [38]. This work addresses representation sharing and task balancing, but it does not explicitly enforce the parent–child geometric consistency required between tree-level and component-level instances in hierarchical 3D orchard segmentation.

Applying existing 3D panoptic-segmentation methods directly to orchard scenes presents a specific challenge: instances are organized hierarchically. Unlike the relatively independent vehicles and pedestrians in urban scenes, an orchard contains multiple fruit trees, and each tree comprises one trunk and several fruits. This relationship is not merely physical inclusion; it also introduces spatial constraints because fruits belonging to the same tree are distributed within the extent of its canopy. Hierarchical panoptic segmentation must therefore provide three outputs. Semantic segmentation assigns a category such as ground, plant/canopy, fruit, trunk, or pole to each point. Standard-instance segmentation distinguishes each fruit and trunk as a fine-grained instance, supporting yield statistics and picking localization. Tree-instance segmentation groups one trunk and all of its fruits into a coarse-grained parent instance. Components belonging to one tree share the same tree ID while retaining different standard-instance IDs.

Existing panoptic-segmentation methods often treat different instance granularities as independent tasks, which creates three limitations. First, they may overlook differences in the required features. Tree-instance segmentation must associate spatially dispersed components, because a trunk and its distant fruits may be several meters apart, and therefore requires large-scale contextual information. By contrast, standard-instance segmentation must distinguish adjacent fruits that may be only a few centimeters apart and therefore requires fine local boundary information. Supplying the same encoder output to all decoders makes it difficult to satisfy both requirements. Second, conventional U-Net skip connections transfer multi-scale encoder features by direct concatenation or addition. The encoder contains information from all categories, whereas the tree decoder focuses on the global structure of a tree and the standard-instance decoder focuses on individual fruit boundaries. Without adaptive selection, skip connections may introduce information that is irrelevant to a particular decoder. Third, independently optimized child- and parent-instance predictions may be hierarchically inconsistent. For example, a fruit can be segmented correctly as an individual instance but assigned to the wrong tree instance.

The resulting failure is specific to orchard management rather than only to generic segmentation accuracy. If fruit and trunk instances are predicted independently from the parent-tree instance, fruits from the same physical tree may be divided among different tree IDs when occlusion, missing points, or canopy gaps weaken their spatial connection to the trunk. Conversely, fruits from neighboring trees may be assigned to the same tree ID when adjacent canopies overlap. In both cases, the fine fruit masks and the total fruit count can appear reasonable, but yield attribution to individual trees becomes incorrect. HOPS and HAPT3D directly expose this requirement by defining hierarchical labels in which each tree instance consists of one trunk and all fruit instances assigned to that tree [8]. This hierarchy makes HAPT3D the closest baseline for our task and motivates hierarchy-aware feature transfer and loss design rather than single-granularity panoptic prediction alone.

HAPT3D [8] introduced hierarchical panoptic segmentation for agricultural scenes and uses a multi-decoder architecture to perform semantic, tree-instance, and standard-instance segmentation simultaneously. However, it transfers information among decoders through direct feature concatenation, without adaptive feature selection, and its loss functions do not explicitly model hierarchical geometric constraints. To address these limitations, we propose HAE-Net, a hierarchy-aware method based on a shared encoder–multi-decoder architecture. HAE-Net models the orchard hierarchy at three stages: task-specific feature generation, adaptive feature transfer, and hierarchical loss design. The main contributions are as follows:

(1) The Hierarchical Feature Enhancement (HFE) module addresses the distinct feature requirements of the three tasks through specialized enhancement paths at the encoder output. A global-context branch combines dilated convolution and global pooling to aggregate dispersed tree components. A multi-scale semantic branch balances contextual information and boundary clarity, while a local-detail branch strengthens the boundary responses needed to distinguish adjacent standard instances. The three paths operate in parallel and provide differentiated initial features to the decoders.
(2) The Cross-Decoder Attention Gate (CDAG) improves feature selection in skip connections. Decoder features serve as gating signals, and the module estimates attention weights across spatial position, feature channel, receptive-field scale, and pixel-level fusion. Each decoder can therefore select the encoder information that is most relevant to its task while suppressing less relevant responses.
(3) The Hierarchical Consistency Loss (HCL) explicitly models the geometric relationship between parent and child instances. For the points belonging to the same ground-truth tree, it constrains the mean predicted standard-instance center to align with the mean predicted tree-instance center. This loss couples the fine-grained instance predictions with their coarse-grained parent assignment.
(4) A comprehensive evaluation on HOPS combines quantitative comparisons and ablation studies on the labeled TLS validation split with qualitative assessment on the TLS, UAV, UGV, and SfM test subsets.

The remainder of the paper is organized as follows. Sec. 2 describes the proposed method, Sec. 3 introduces the dataset, Sec. 4 presents the experimental results and analysis, and Sec. 5 summarizes the study and its conclusions.

## 2 Method

HAE-Net is designed for hierarchical panoptic segmentation in orchards. Given an input point cloud $P={\left\{\left(p_{i},c_{i}\right)\right\}}_{i=1}^{N}$, where $p_{i}\in R^{3}$ denotes the 3D coordinates of point *i* and $c_{i}\in R^{3}$ denotes its RGB features, the network predicts three labels for each point. The semantic label *s*_*i*_ ∈ {1, 2, …, *K*} identifies one of *K* categories, including the “stuff” classes ground, plant, and pole and the “things” classes fruit and trunk. The standard-instance label $o_{i}\in Z^{+}$ distinguishes individual fruits and trunks at the fine-grained level. At the coarser level, the tree-instance label $t_{i}\in Z^{+}$ identifies the parent tree of each component. One tree instance contains a trunk and all fruits assigned to it, so these components share *t*_*i*_ but retain distinct *o*_*i*_ values. HAE-Net models this inclusion relationship across its three outputs.

HAE-Net uses the shared encoder–multi-decoder architecture depicted in Fig. 1(a). The input point cloud is voxelized at a fixed resolution and represented as a sparse tensor, from which the shared sparse-convolutional encoder extracts multi-scale features. HFE transforms the encoder output into separate initial representations for the semantic, tree-instance, and standard-instance decoders. As spatial resolution is recovered, CDAGs regulate the encoder features passed through the skip connections; features from the preceding decoder provide the gating signal. Semantic, standard-instance, tree-instance, and HCL terms jointly optimize the three outputs. At inference time, clustering the predicted centers yields instance labels, which are combined with the semantic predictions.

**Fig. 1:**
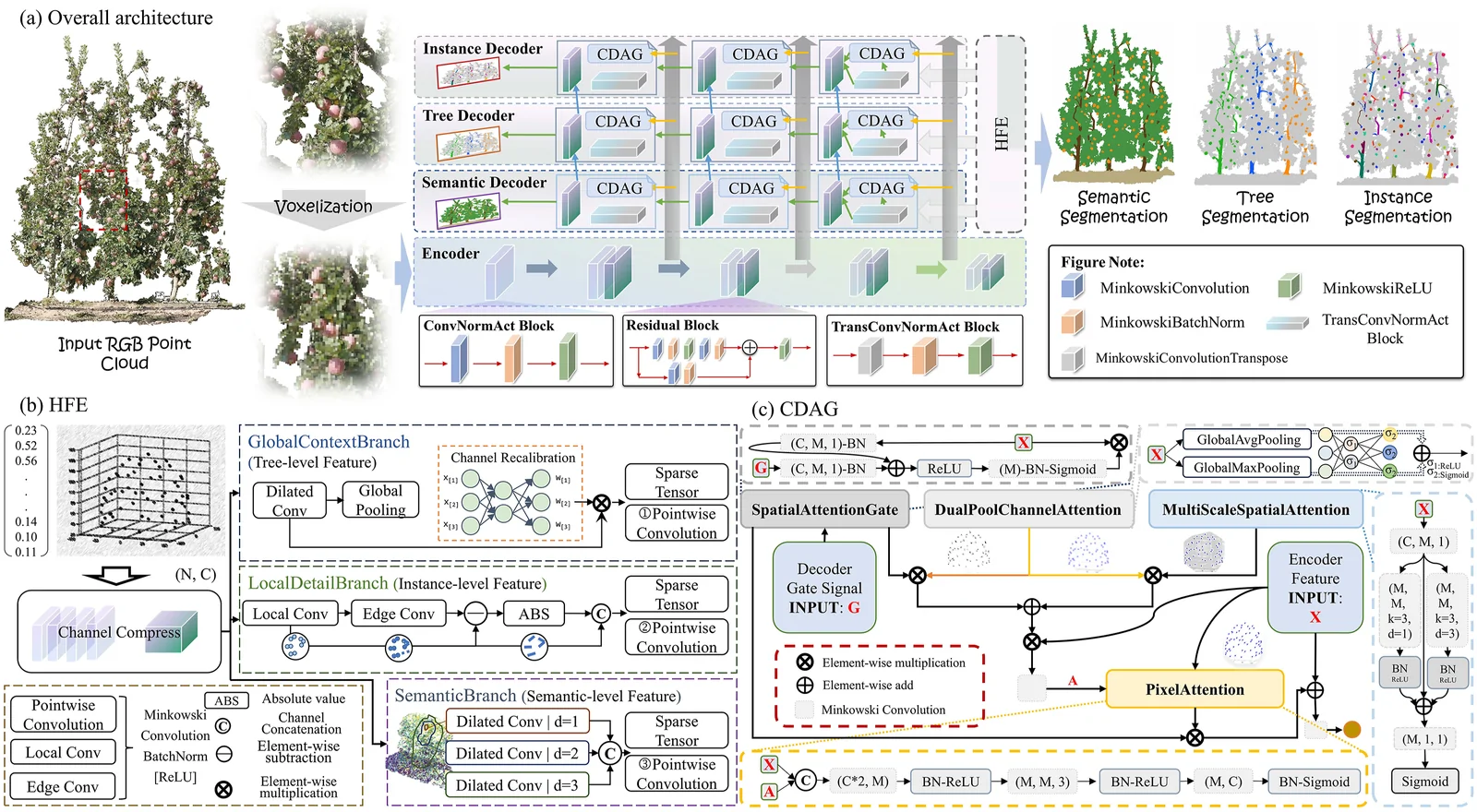
Overview of HAE-Net and its principal modules. (a) Overall architecture of the shared encoder and three task-specific decoders. (b) Hierarchical Feature Enhancement (HFE) module. (c) Cross-Decoder Attention Gate (CDAG).

### 2.1 Hierarchical Feature Enhancement (HFE) module

Conventional multi-decoder architectures often provide the same encoder output to all decoders. However, the three tasks in hierarchical panoptic segmentation require different types of information. Tree-instance segmentation must associate spatially dispersed components with the same parent tree. Because a fruit may be several meters from the trunk, this task requires a large receptive field and long-range contextual information while remaining resistant to local noise. Semantic segmentation assigns a class to every point, including points near category boundaries such as the boundary between plant and fruit. It therefore requires sufficient semantic context for classification as well as localized boundary information. Standard-instance segmentation must separate adjacent objects of the same category, such as fruits only a few centimeters apart. This task depends on high-resolution local detail and strong responses near instance boundaries. HFE generates a separate initial representation for each decoder to address these differences.

The HFE module is shown in Fig. 1(b). Unlike a design that only assigns different dilation rates to the decoders, HFE applies a task-specific enhancement strategy to each branch.

(1) Global-context branch for the tree-instance decoder. This branch aggregates dispersed tree components by using a large-dilation convolution to expand the receptive field. Global average pooling summarizes scene-level information, and a channel-recalibration mechanism emphasizes feature channels that respond to global tree structure:

(1) $$W_{ch}=\sigma \left(\text{MLP}\left(\text{GAP}\left(\phi_{d=4}\left(F_{e}\right)\right)\right)\right)$$

(2) $$F_{\mathrm{tree}}=W_{ch}⊙\phi_{d=4}\left(F_{e}\right)$$

where *ϕ*_*d*=4_ denotes a sparse convolution with dilation rate 4, GAP denotes global average pooling, and $W_{ch}\in R^{C}$ contains the learned channel weights. The recalibrated output **F**_*tree*_ combines the expanded receptive field with channel-specific global context.

(2) Multi-scale semantic branch for the semantic decoder. Semantic classification requires contextual information without losing category boundaries. Three convolutional branches with small, medium, and large dilation rates capture complementary spatial scales. The small-dilation branch preserves local boundary detail, the medium-dilation branch provides the main semantic representation, and the large-dilation branch supplies broader context. Their outputs are concatenated and fused as follows:

(3) $$F_{\mathrm{sem}}=\psi \left(\left[\phi_{d=1}\left(F_{e}\right);\phi_{d=2}\left(F_{e}\right);\phi_{d=3}\left(F_{e}\right)\right]\right)$$

where [⋅; ⋅] denotes feature concatenation and *ψ* is a 1 × 1 × 1 convolution used to fuse the three scale-specific features.

(3) Local-detail branch for the standard-instance decoder. This branch emphasizes differences near local instance boundaries. A small-dilation convolution retains spatial detail, while a boundary-enhancement operation calculates the difference between the local feature and an additional transformation of that feature:

(4) $$F_{\mathrm{edge}}=|\phi_{d=1}\left(F_{e}\right)-\phi^{\prime }\left(\phi_{d=1}\left(F_{e}\right)\right)|$$

(5) $$F_{\mathrm{inst}}=\psi \left(\left[\phi_{d=1}\left(F_{e}\right);F_{\mathrm{edge}}\right]\right)$$

where *ϕ*′ is an additional 3 × 3 × 3 convolution. The absolute difference **F**_*edge*_ highlights locations where the two local transformations produce different responses, in a manner analogous to an edge-sensitive residual. This boundary feature is concatenated with the local feature to obtain **F**_*inst*_.

Different receptive-field sizes alone do not satisfy the three segmentation tasks. Changing only the dilation rate alters the spatial support of a convolution but does not tailor the transformation to the information needed by each decoder. The tree branch therefore couples an enlarged receptive field with global pooling and channel recalibration. The semantic branch fuses several receptive fields instead of selecting one, while the standard-instance branch explicitly derives an edge-sensitive difference feature. HFE runs these operations in parallel and sends each output to its corresponding decoder: global aggregation supports tree-instance segmentation, context–boundary balance supports semantic segmentation, and local boundary sensitivity supports standard-instance segmentation.

### 2.2 Cross-Decoder Attention Gate (CDAG)

U-Net-style encoder–decoder architectures transfer multi-scale encoder features through skip connections to recover spatial detail lost during downsampling. Direct concatenation is less suitable for hierarchical panoptic segmentation because the encoder mixes information from all categories, whereas each decoder has a different target. The tree-instance decoder emphasizes the global structure of a complete tree; the standard-instance decoder instead emphasizes the local boundaries of individual fruits and trunks. Spatial positions and feature channels also contribute differently to the three tasks, so unfiltered skip features may include responses that a particular decoder does not need. Attention gating uses a decoder signal to select relevant encoder features [39]. SCSA [40] jointly models spatial and channel attention, while the Content-Guided Attention Fusion module in DEA-Net [41] combines spatial, channel, and pixel-level attention for adaptive feature fusion. CDAG adapts these ideas to feature transfer among the hierarchical decoders and adds explicit multi-scale selection.

At each skip connection, CDAG takes encoder features $F_{\mathrm{enc}}\in R^{N\times C}$ and upsampled decoder features $F_{\mathrm{dec}}\in R^{N\times C}$ as inputs (Fig. 1(c)). The decoder representation acts as a gating signal for selection by spatial position, feature channel, receptive-field scale, and pixel-level fusion.

The four attention dimensions are designed to answer different selection questions rather than to repeat the same operation. Spatial attention asks which points in the skip feature should be emphasized for the current decoder. Channel attention asks which latent feature channels should be retained across the point cloud. Multi-scale attention asks which receptive-field scale is more suitable at each point, distinguishing local fruit-boundary evidence from broader canopy or tree-structure context. Pixel-level fusion then resolves the combined spatial, channel, and scale cues into a point-wise modulation before the residual connection restores the original encoder feature. Thus, spatial and multi-scale attention both produce point-dependent weights, but they are not redundant: the former is decoder-conditioned and controls task-relevant locations, whereas the latter is encoder-derived and controls the local–contextual scale preference at those locations.

Spatial attention gate. Points at different spatial positions contribute differently to each task. Following the gating formulation of Attention U-Net [39], the decoder features guide the point-wise selection of encoder features. The CDAG at the tree-instance decoder uses features from the semantic decoder as its gating signal, while the CDAG at the standard-instance decoder uses features from the tree-instance decoder:

(6) $$A_{\mathrm{spatial}}=\sigma \left(W_{\psi }\left(\text{ReLU}\left(W_{\mathrm{enc}}F_{\mathrm{enc}}+W_{\mathrm{dec}}F_{\mathrm{dec}}\right)\right)\right)$$

where $A_{\mathrm{spatial}}\in R^{N\times 1}$ contains one spatial weight for each point and controls the contribution of that location to the transferred encoder feature.

Dual-pooling channel attention. Feature channels encode different response patterns, and their relevance can vary across tasks. Global average pooling summarizes the overall response of each channel, whereas global max pooling captures its strongest response. Drawing on the complementary average- and max-pooling descriptors used in CBAM [42], CDAG combines both statistics to estimate channel importance:

(7) $$A_{\mathrm{channel}}=\sigma \left(\text{MLP}\left(\text{GAP}\left(F_{\mathrm{enc}}\right)\right)\right)+\sigma \left(\text{MLP}\left(\text{GMP}\left(F_{\mathrm{enc}}\right)\right)\right)$$

where $A_{\mathrm{channel}}\in R^{C}$ contains the channel weights applied to the encoder representation.

Multi-scale spatial attention. Orchard objects vary substantially in spatial extent: individual fruits occupy small local regions, whereas a tree canopy may span several meters. A single receptive field may not represent both scales effectively. Following the multi-scale spatial-attention principle used in CPCA [43], CDAG uses two convolutional branches with different dilation rates to capture local detail and broader context:

(8) $$A_{\mathrm{multi}}=\sigma \left(\text{Conv}\left(\phi_{d=1}\left(F_{\mathrm{enc}}\right)+\phi_{d=3}\left(F_{\mathrm{enc}}\right)\right)\right)$$

where *ϕ*_*d*=*k*_ denotes a 3 × 3 × 3 convolution with dilation rate *k*, and $A_{\mathrm{multi}}\in R^{N\times 1}$ denotes the resulting multi-scale spatial weights. The small-dilation branch retains local information, while the large-dilation branch captures a wider spatial context.

Pixel-level attention fusion. The spatial, channel, and multi-scale mechanisms describe feature importance from complementary perspectives. CDAG uses a progressive fusion strategy [41]: it first combines the spatial and multi-scale weights with the channel weights and then estimates a point-wise fusion tensor from the weighted encoder features.

(9) $$P_{1}=\left(A_{\mathrm{spatial}}\cdot A_{\mathrm{channel}}\right)+\left(A_{\mathrm{multi}}\cdot A_{\mathrm{channel}}\right)$$

(10) $$P_{2}=\sigma \left(\text{Conv}\left(\left[F_{\mathrm{enc}};\psi \left(P_{1}⊙F_{\mathrm{enc}}\right)\right]\right)\right)$$

where *ψ* is a 1 × 1 × 1 convolution used to transform the weighted feature. A residual connection then retains the original encoder representation while adding the attention-modulated response:

(11) $${\hat{F}}_{\mathrm{enc}}=F_{\mathrm{enc}}+P_{2}⊙\left(A_{\mathrm{spatial}}⊙F_{\mathrm{enc}}\right)$$

where [⋅; ⋅] denotes feature concatenation and ⊙ denotes element-wise multiplication.

Operationally, CDAG first forms two channel-reweighted spatial descriptors: one from the decoder-conditioned spatial gate and the other from the encoder-derived multi-scale gate. These descriptors are summed in **P**_1_, so a point receives a high preliminary response only when its location, feature channels, and scale context are jointly relevant. The convolution in **P**_2_ then learns a fused point-level weight from the original encoder feature and this preliminary response. The final residual form prevents the gate from discarding the skip feature completely; instead, CDAG adds an attention-modulated correction to the original encoder representation. This design allows boundary-sensitive points, tree-level contextual regions, and semantically informative areas to be emphasized differently by different decoders.

Each decoder uses CDAG at every skip-connection level. HFE separates the three initial representations at the encoder output; CDAG subsequently filters the encoder information transferred during upsampling. The spatial and multi-scale terms vary across points, whereas the channel term changes the contribution of latent feature channels. Pixel-level fusion resolves their combined effect before the residual connection restores the original encoder signal. Task-specific selection thus occurs both before decoding and as spatial resolution is reconstructed, without discarding the shared representation altogether.

### 2.3 Hierarchical consistency loss (HCL)

To address the gap identified in the Introduction, HCL complements the semantic and instance losses with an explicit geometric constraint between tree-level parent instances and their component-level child instances. The orange circles in Fig. 2 mark the ground-truth centers of the fruit and trunk instances belonging to one tree. Each point produces two predicted centers. Light-blue asterisks represent the standard-instance centers predicted for individual fruits or trunks, and light-purple crosses represent the centers predicted for the complete tree instance. Their respective means, ${\bar{e}}_{T_{k}}^{\mathrm{inst}}$ and ${\bar{e}}_{T_{k}}^{\mathrm{tree}}$, are shown by the dark-blue asterisk and dark-purple cross.

**Fig. 2:**
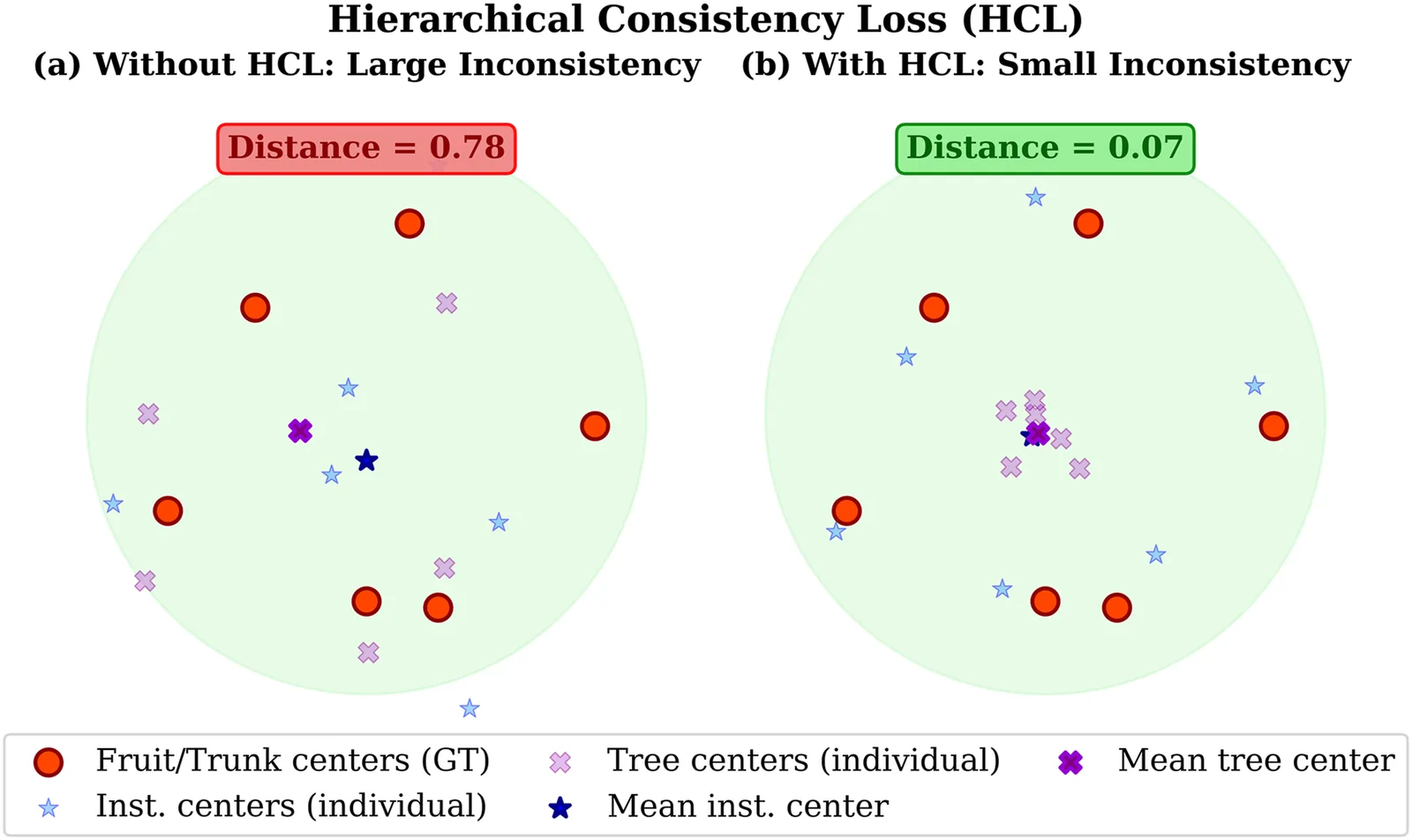
Illustration of the hierarchical consistency loss (HCL).

HCL does not require fruits to follow a geometrically uniform distribution around the tree. Its first-order formulation nevertheless assumes that the point-averaged standard-instance and tree-instance center fields provide sufficiently representative summaries of the same annotated tree. In the HOPS annotation [8], one tree instance contains the trunk and all fruit instances assigned to the same physical tree. The two decoders therefore describe the same parent structure at different granularities, and their aggregated predicted centers should not drift far apart during training. The constraint is used as a soft regularizer, controlled by *λ*, to reduce disagreement between the two center fields; it is not used as an inference rule and does not force all component centers to collapse to one point. The left side of Fig. 2 illustrates an example without the HCL constraint, where the distance between the two means is 0.78. On the right, the distance is 0.07 after the constraint is applied. HCL formalizes this geometric prior by penalizing the distance between the two mean centers during training.

Let $o_{i}^{\mathrm{inst}}\in R^{3}$ and $o_{i}^{\mathrm{tree}}\in R^{3}$ denote the offsets predicted by the standard-instance and tree-instance decoders, respectively. The corresponding predicted centers are

(12) $$e_{i}^{\mathrm{inst}}=p_{i}+o_{i}^{\mathrm{inst}},\;e_{i}^{\mathrm{tree}}=p_{i}+o_{i}^{\mathrm{tree}}$$

For each training sample, let $T={\left\{T_{k}\right\}}_{k=1}^{N_{\text{tree}}}$ denote the set of tree instances, where *N*_tree_ is the number of trees and *T*_*k*_ is the point set belonging to the trunk and fruits of the *k*-th tree. The hierarchical consistency loss is

(13) $$L_{\mathrm{HCL}}=\frac{1}{N_{\text{tree}}}\overset{N_{\text{tree}}}{\underset{k=1}{\sum }}{\left\|{\bar{e}}_{T_{k}}^{\mathrm{inst}}-{\bar{e}}_{T_{k}}^{\mathrm{tree}}\right\|}_{2}^{2}$$

Let *N*_*k*_ denote the number of points in *T*_*k*_. The mean predicted standard-instance and tree-instance centers for all points associated with *T*_*k*_ are

(14) $${\bar{e}}_{T_{k}}^{\mathrm{inst}}=\frac{1}{N_{k}}\underset{i\in T_{k}}{\sum }e_{i}^{\mathrm{inst}},\;{\bar{e}}_{T_{k}}^{\mathrm{tree}}=\frac{1}{N_{k}}\underset{i\in T_{k}}{\sum }e_{i}^{\mathrm{tree}}$$

The loss constrains the point-averaged standard-instance center field within one annotated tree to remain consistent with the point-averaged tree-instance center field. Geometrically, standard-instance centers correspond to individual fruit or trunk instances, whereas each tree-instance center corresponds to a parent tree-level instance, defined as one trunk together with all fruits assigned the same tree ID. The arithmetic mean is used as a simple differentiable summary of the predicted center field for each annotated tree. Its role is to regularize the relative displacement between the child-level and parent-level predictions, not to represent the horticultural center of fruit production. Because both predictions describe different granularities of the same physical tree, their aggregated centers should be spatially aligned. This constraint encourages point-wise offset prediction to preserve fine instance boundaries while accounting for the broader relationship between each component and its parent tree. This formulation therefore maintains fine-grained segmentation precision while strengthening coarse-grained grouping integrity. The applicable scope of this formulation is therefore the common case in which the visible trunk and fruit observations provide a sufficiently representative set of components for a tree-level regularizer. Strongly one-sided fruit visibility, severe partial scans, or heavily occluded canopies can bias the arithmetic mean and may require a more robust center estimator. In the example in Fig. 2, the reduction from 0.78 to 0.07 illustrates the intended geometric effect of HCL.

### 2.4 Network training and inference

The overall training objective contains four terms:

(15) $$L=L_{\mathrm{sem}}+L_{\mathrm{inst}}+L_{\mathrm{tree}}+\lambda L_{\mathrm{HCL}}$$

Here, $L_{\mathrm{sem}}$ is the cross-entropy loss for semantic segmentation; $L_{\mathrm{inst}}$ and $L_{\mathrm{tree}}$ are Lovász hinge losses for standard and tree instances, respectively [44]; and *λ* weights the hierarchical-consistency term. The semantic loss is $L_{\mathrm{sem}}=-\frac{1}{N}\sum_{i=1}^{N}\sum_{k=1}^{K}y_{ik}\log\left({\hat{y}}_{ik}\right)$, where *y*_*ik*_ is the ground-truth indicator for class *k* at point *i* and ${\hat{y}}_{ik}$ is the predicted probability of that class. The standard-instance loss is $L_{\mathrm{inst}}=\frac{1}{\left|C\right|}\sum_{j=1}^{\left|C\right|}\text{Lovász}\left(F_{j},G_{j}\right)$, where C is the set of valid instances, **F**_*j*_ is the predicted soft mask derived from the offsets, and **G**_*j*_ is the corresponding ground-truth mask. The tree-instance loss is defined analogously for tree-level masks. This joint objective optimizes point-wise semantic classification, the two instance granularities, and their geometric relationship.

During inference, a forward pass produces semantic predictions and two sets of instance-offset vectors. For points classified as “things,” the corresponding predicted centers are clustered using HDBSCAN [20]. Applying this procedure to both sets of centers generates standard-instance and tree-instance labels. These assignments are then combined with the semantic labels to form the hierarchical panoptic output.

## 3 Dataset

Experiments are conducted on the Hierarchical Orchard Panoptic Segmentation (HOPS) dataset [8], which was collected in an apple orchard near Bonn, Germany. HOPS contains point clouds from four acquisition sources. The terrestrial-laser-scanner (TLS) subset contains high-precision, high-density point clouds and provides the training, validation, and part of the test data. The uncrewed-aerial-vehicle (UAV) subset was acquired from aerial viewpoints at 45°, 90°, and 135°. The uncrewed-ground-vehicle (UGV) subset was captured using a RealSense D435i depth camera mounted on a ground platform. The Structure-from-Motion (SfM) subset was reconstructed from the Fuji-SfM data [6,16]. The five data splits are summarized in Supplementary Table S1. Training and validation use only TLS data, whereas the four test subsets represent TLS, UAV, UGV, and SfM acquisition conditions. In the public release, semantic, standard-instance, and tree-instance annotations are available for the training and validation splits, whereas the TLS, UAV, UGV, and SfM test subsets contain point clouds without publicly accessible ground-truth labels.

HOPS defines six semantic labels, including the ignored void label, as listed in Supplementary Table S2. Ground, plant, and pole are “stuff” classes, whereas fruit and trunk are “things” classes.

The dataset also provides hierarchical annotations in which each tree-level instance comprises one trunk and all fruits assigned to that tree. Each labeled point-cloud sample contains point coordinates (positions), RGB values (colors), one of six semantic labels (semantic), a binary hierarchical semantic label that distinguishes foreground from background (semantic_h), a standard-instance label (instance), and a tree-instance label (instance_h). The accompanying metadata include the sensor type (sensor) and file name (filename). The labeled TLS example in Fig. 3(a) shows, from left to right, the RGB point cloud, semantic annotation, foreground–background annotation, standard-instance annotation, and tree-instance annotation. Fruits and trunks form the foreground in the binary annotation; ground, canopy, and the other “stuff” classes form the background. The differences in viewpoint, density, and reconstruction across the SfM, TLS, UAV, and UGV subsets are illustrated in Fig. 3(b).

**Fig. 3:**
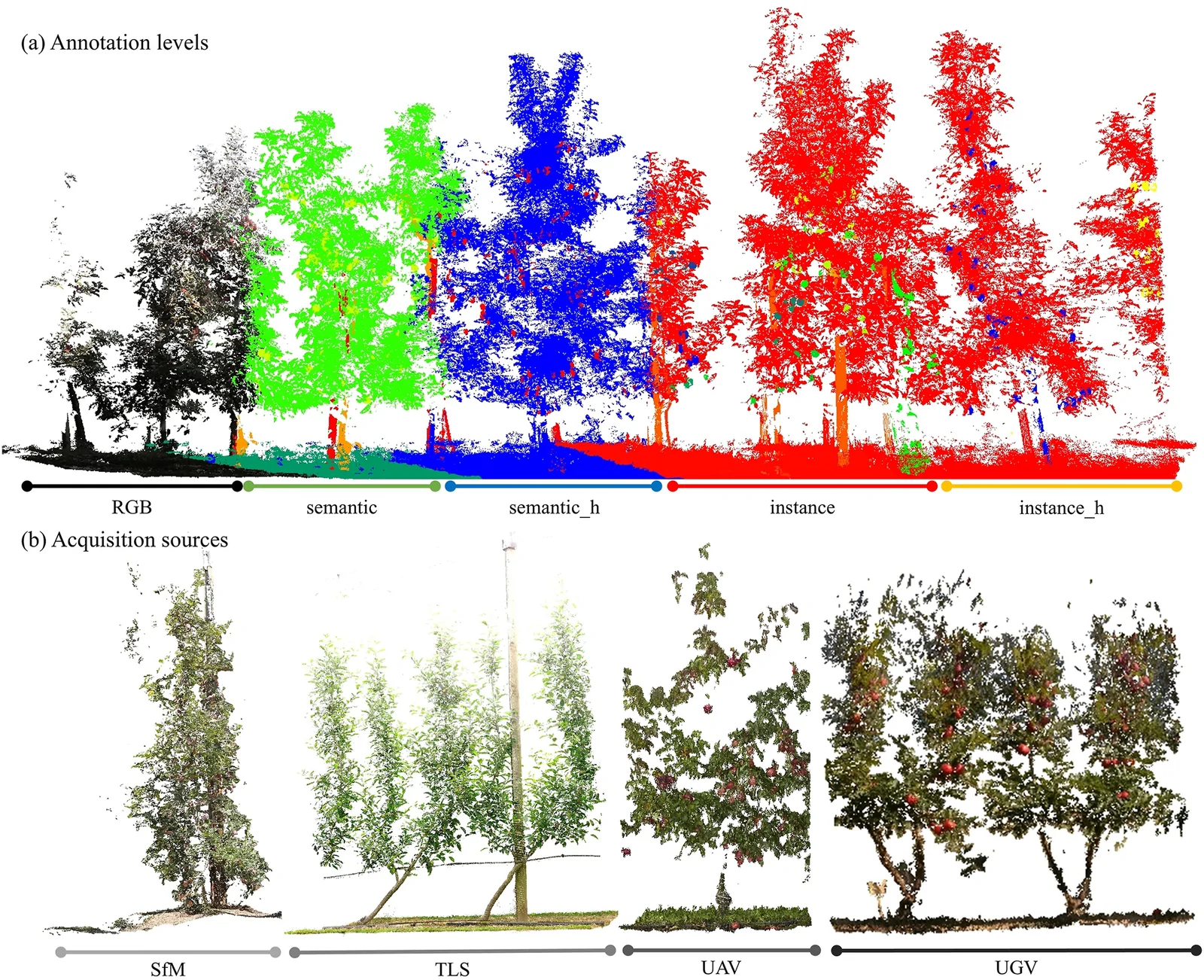
Overview of the HOPS data. (a) A labeled TLS point cloud showing the RGB input and the semantic, foreground–background, standard-instance, and tree-instance annotations. (b) Representative RGB point clouds from the SfM, TLS, UAV, and UGV acquisition sources.

## 4 Experiments and analysis

We evaluate HAE-Net on the HOPS dataset through quantitative comparisons, ablation studies, and qualitative visualization. The quantitative experiments compare HAE-Net with five representative methods: MaskPLS [45], ForestPS [46], P3Former [47], SuperCluster [48], and HAPT3D [8]. HAPT3D serves as the task-specific hierarchical baseline, while the other four methods provide additional comparisons. The experiments also assess the contributions of HFE, CDAG, and HCL using mIoU, PQ, PQ_T_, and mPQ. The public HOPS release provides ground-truth annotations for the training and validation splits but withholds the semantic, standard-instance, and tree-instance labels for all four test subsets. Accordingly, all reported quantitative comparisons and ablation studies are conducted on the labeled TLS validation split. Predictions on the TLS, UAV, UGV, and SfM test subsets are used only for qualitative inspection, and no quantitative conclusion about cross-sensor performance is drawn from them.

### 4.1 Experimental parameters and evaluation metrics

The experiments were implemented in PyTorch 1.12.1 with CUDA 11.3 and run on Ubuntu 18.04 using an Intel Xeon Gold 6226R CPU and an NVIDIA A30 GPU. Training used AdamW with cosine-annealing learning-rate scheduling and a 10-epoch warm-up period. Random scaling, rotations about all three axes, shearing, and subsampling varied the geometry and point distribution, while contrast, brightness, hue, and saturation perturbations changed the color input. The complete configuration in Supplementary Table S3 also specifies the 3-mm voxel resolution, learning-rate range, early-stopping criterion, maximum number of epochs, batch size, random seed, and augmentation ranges.

Hierarchical panoptic segmentation must be evaluated at the semantic level and at both instance granularities. We therefore report the following four metrics:

(1) Mean Intersection over Union (mIoU) is the class-averaged intersection over union for the five evaluated semantic categories, with the void label excluded. It measures the accuracy of point-wise semantic classification across both “things” and “stuff” classes.
(2) Panoptic Quality (PQ) evaluates standard-instance segmentation for the “things” classes and semantic segmentation for the “stuff” classes. It is defined as PQ = SQ ×RQ, where Segmentation Quality (SQ) measures the overlap of matched segments and Recognition Quality (RQ) measures the accuracy with which segments are recognized.
(3) Tree-level Panoptic Quality (PQ_T_) applies the panoptic-quality calculation at the parent-instance level. Unlike standard PQ, which treats individual fruits and trunks as separate instances, PQ_T_ treats one trunk and all fruits assigned to the same tree as a single tree instance. It therefore measures whether the components of each tree are correctly grouped at the coarser hierarchical level.
(4) Mean Panoptic Quality (mPQ) is the arithmetic mean of PQ and PQ_T_. It provides an aggregate measure of panoptic performance across the standard-instance and tree-instance granularities.

### 4.2 Comparative experiments

We compare HAE-Net with five representative methods. MaskPLS [45] extends query-based mask classification to 3D point clouds. DigiForests [46] provides a MinkUNet-based panoptic model [13], referred to here as ForestPS. P3Former [47] is a recent position-guided transformer for point-cloud panoptic segmentation, and SuperCluster [48] formulates scalable 3D panoptic segmentation as superpoint-graph clustering. HAPT3D [8] is the task-specific hierarchical baseline and connects a shared encoder to multiple decoders by direct feature concatenation. Among these methods, HAPT3D and HAE-Net are hierarchical multi-task models that produce semantic, standard-instance, and tree-instance predictions in a single training run. MaskPLS, ForestPS, P3Former, and SuperCluster are single-granularity 3D panoptic methods; therefore, they are adapted to HOPS by training separate models for standard-instance and tree-instance labels. On the labeled HOPS validation split, HAE-Net attains the highest value for every metric. Its mIoU increases from 61.82 to 71.63 relative to HAPT3D, while mPQ increases from 48.53 to 53.96, gains of 9.81 and 5.43 percentage points, respectively.

This table separates the task-specific baseline from the adapted comparisons. HAPT3D is the closest baseline because it is designed for the same hierarchical orchard panoptic task and generates the two instance granularities jointly. The four single-granularity methods provide additional point-cloud panoptic comparisons, but their hierarchical metrics require two independent training runs. For these methods, mIoU is computed from the semantic predictions of the standard-instance run, PQ is obtained from the standard-instance run, PQ_T_ is obtained from the tree-instance run, and mPQ is computed uniformly as the arithmetic mean of PQ and PQ_T_.

Fig. 4 provides a qualitative comparison on two validation samples. Each sample includes the ground-truth annotation and the predictions of MaskPLS, ForestPS, P3Former, SuperCluster, HAPT3D, and HAE-Net. The three result columns are explicitly labeled as Semantic, Tree-level, and Instance segmentation, corresponding to semantic classes, parent tree instances, and standard fruit/trunk instances, respectively. This layout separates the proposed method from the competing methods and allows the three prediction levels to be inspected side by side. In the displayed cases, HAE-Net follows the annotated semantic boundaries more closely, retains more small fruit regions, and separates adjacent tree instances that competing methods may miss or merge. Although these examples are not a substitute for aggregate evaluation, they show the kinds of errors reflected in Table 1.

**Fig. 4:**
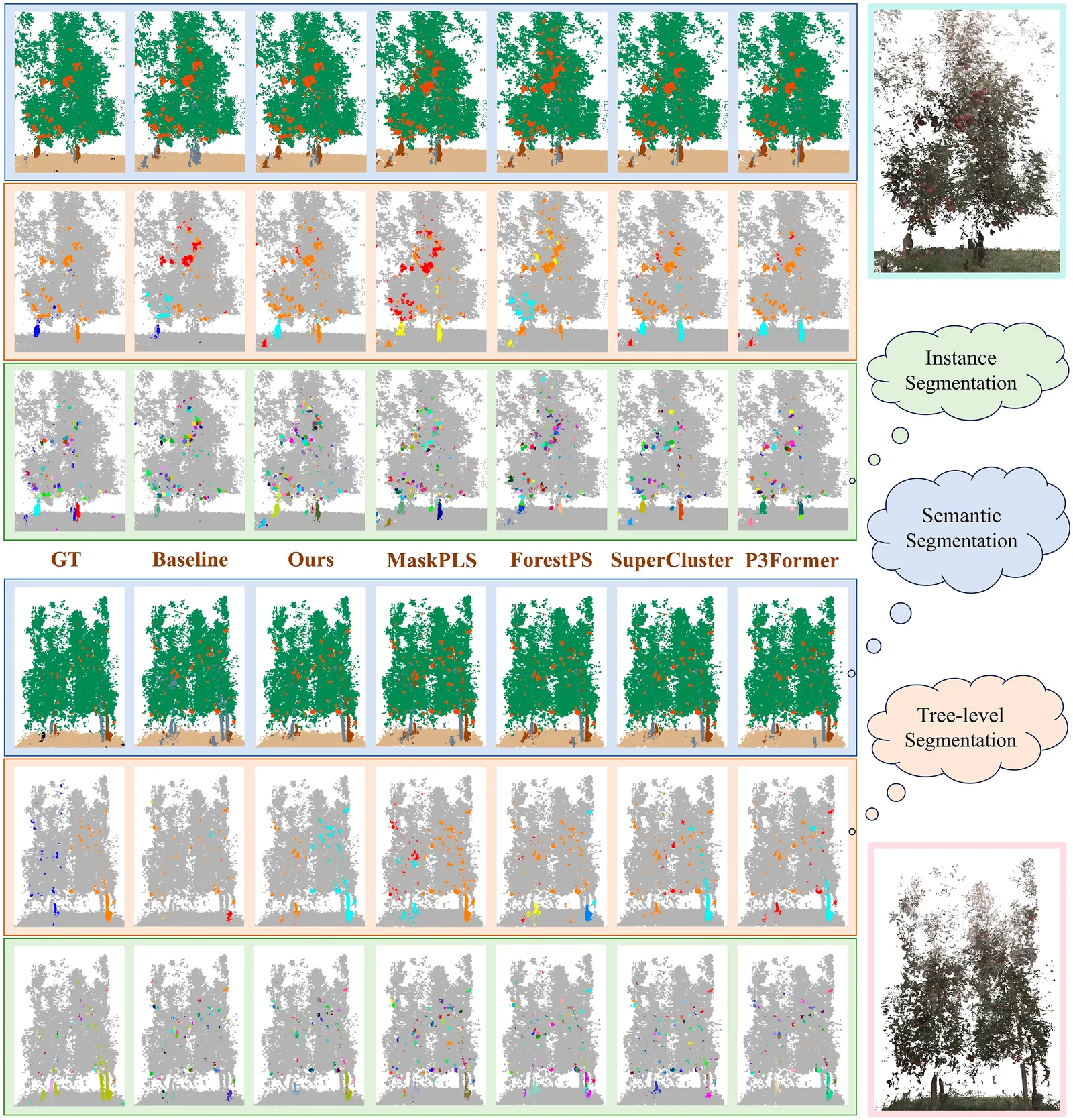
Qualitative comparison of segmentation results on the validation set. Columns show the ground truth and predictions of MaskPLS, ForestPS, P3Former, SuperCluster, HAPT3D, and HAE-Net; rows show Semantic, Tree-level, and Instance segmentation results.

**Table 1:** Quantitative comparison on the HOPS validation set.

Method	Type	Runs	mIoU *↑*	PQ *↑*	PQ_T_*↑*	mPQ *↑*
MaskPLS	Single-granularity	2	27.63	40.39	46.72	43.56
ForestPS	Single-granularity	2	56.71	49.46	23.64	36.55
P3Former	Single-granularity	2	63.24	49.52	50.25	49.89
SuperCluster	Single-granularity	2	24.77	32.69	37.81	35.25

HAPT3D	Hierarchical multi-task	1	61.82	47.73	49.33	48.53
**Ours**	Hierarchical multi-task	1	71.63	54.55	53.37	53.96

Because accurate and efficient 3D point-cloud analysis is a key concern in recent 3D plant phenomics research [49], we also report a performance–efficiency comparison in Table 2. Training time denotes the total wall-clock time required to obtain the selected checkpoint or checkpoints under the validation protocol. For single-granularity methods, the reported total includes both the standard-instance and tree-instance training runs.

**Table 2:** Performance–efficiency trade-off on the HOPS validation set.

Method	Params (M)	Training runs	Total training time (h)	Inference time/sample (s)	Peak GPU memory (GB)
MaskPLS	1.8	2	23	4.00	8.9
ForestPS	1.5	2	25	1.80	8.5
P3Former	77.4	2	37	6.30	22.0
SuperCluster	0.2	2	9	1.10	4.2
HAPT3D	19.4	1	14	2.00	14.3
Ours	18.3	1	13	1.84	12.9

The efficiency results further clarify the fairness of the comparison. P3Former obtains the strongest mPQ among the adapted single-granularity comparison methods, but it requires 77.4 M parameters, two training runs, 37 h total training time, 6.30 s per validation sample, and 22.0 GB peak GPU memory. SuperCluster is the most lightweight comparison method, but its mPQ is 35.25. Compared with the task-specific hierarchical baseline HAPT3D, HAE-Net improves mPQ from 48.53 to 53.96 while using fewer parameters (18.3 M versus 19.4 M), shorter total training time (13 h versus 14 h), lower inference time (1.84 s versus 2.00 s per sample), and lower peak GPU memory (12.9 GB versus 14.3 GB). Thus, the main comparison with HAPT3D remains the most direct task-level comparison, and the expanded comparison set shows that HAE-Net provides a favorable accuracy–efficiency trade-off under the HOPS validation protocol.

To assess whether the main comparison is stable with respect to random initialization and stochastic training, we trained HAPT3D and HAE-Net using six random seeds: 0, 42, 2026, 3141, 27182, and 142857. This repeated-seed analysis focuses on the main HAPT3D–HAE-Net comparison. The single-run comparisons and ablation studies use seed 42, while Table 3 reports the mean, standard deviation, paired mean improvement, and exact two-sided Wilcoxon signed-rank test across the six seed pairs.

**Table 3:** Repeated-seed main comparison on the HOPS validation set.

Method	mIoU *↑*	PQ *↑*	PQ_T_*↑*	mPQ *↑*
HAPT3D	61.83 ±0.18	47.65 ±0.19	49.32 ±0.08	48.48 ±0.12
Ours	71.67 ±0.09	54.57 ±0.03	53.37 ±0.03	53.97 ±0.01

Paired improvement	+9.84 ±0.11	+6.92 ±0.20	+4.06 ±0.06	+5.49 ±0.12
Wilcoxon *p*	0.031	0.031	0.031	0.031

The repeated-seed results show the same direction of improvement for every seed and every metric. The mean mIoU increases from 61.83 ±0.18 to 71.67 ±0.09, and the mean mPQ increases from 48.48 ±0.12 to 53.97 ±0.01. The paired improvements are larger than the corresponding run-to-run standard deviations, and the exact Wilcoxon test gives *p* = 0.031 for all four metrics. These results do not remove the limitation that the validation split contains only 18 labeled samples, but they indicate that the observed advantage over HAPT3D is consistent across random seeds rather than being driven by a single training run.

### 4.3 Ablation studies

To evaluate the contribution of each proposed component, all ablation studies were conducted on the HOPS dataset using the same evaluation protocol as the comparative experiments. The baseline for these studies is HAPT3D, and modules are added individually or in combination while the remaining training settings are held fixed.

The module ablation in Table 4 separates the contributions of HFE, CDAG, and HCL and then tests them in combination. When used alone, HFE, CDAG, and HCL raise mPQ from the baseline value of 48.53 to 50.80, 51.72, and 50.52, respectively. Their effects differ across the individual metrics. HFE produces its larger gains in mIoU and standard PQ; CDAG improves PQ and mPQ through task-adaptive feature selection; HCL has its clearest effect on PQ_T_ by constraining the hierarchical relationship. Combining HFE with CDAG raises PQ_T_ to 51.20, and the complete model reaches 71.63 mIoU, 54.55 PQ, 53.37 PQ_T_, and 53.96 mPQ. The slight PQ_T_ decrease produced by HFE alone is analyzed with reference to Supplementary Table S4.

**Table 4:** Module contribution analysis.

HFE	CDAG	HCL	mIoU *↑*	PQ *↑*	PQ_T_*↑*	mPQ *↑*
✗	✗	✗	61.82	47.73	49.33	48.53
✓	✗	✗	65.37	52.37	49.23	50.80
✗	✓	✗	66.74	53.31	50.13	51.72
✗	✗	✓	65.76	51.03	50.01	50.52
✓	✓	✗	67.94	53.77	51.20	52.49
✓	✓	✓	71.63	54.55	53.37	53.96

#### 4.3.1 Ablation of the HFE module

Three ways of transforming the encoder output are compared in Supplementary Table S4. “Shared Features (No HFE)” passes the same representation to all decoders. “Only Dilation Differences” introduces decoder-specific convolutional branches that differ in dilation rate but omit the task-oriented enhancement operations. “Complete Specialized Design” uses the full HFE module: global-context aggregation with channel recalibration for the Tree Decoder, multi-scale fusion for the Semantic Decoder, and local-boundary enhancement for the Instance Decoder.

To further test whether the three HFE branches are necessary, we perform a branch-level ablation while keeping CDAG and HCL disabled. The dilation-only design is used as the controlled baseline because it preserves decoder-specific convolutional branches but removes the task-oriented enhancement operations. The global-context (GC), multi-scale semantic (MS), and local-detail (LD) branches are then enabled individually and in combination, as shown in Supplementary Table S5.

The branch-level results support the task-oriented HFE design, but the effects are metric-specific. Enabling only the GC branch increases PQ_T_ from 48.95 to 49.18, the largest single-branch gain on the tree-level metric, while its changes in mIoU and PQ are small. In the leave-one-branch-out comparison, adding GC to the MS + LD configuration raises PQ_T_ from 48.96 to 49.23, a 0.27-point improvement, confirming that global context mainly benefits tree-level grouping. The MS branch has the clearest effect on semantic segmentation: removing MS from the GC + LD configuration lowers mIoU from 65.37 to 64.99, whereas the single MS branch increases mIoU from 64.88 to 65.24. The LD branch mainly affects standard panoptic quality and the hierarchical aggregate. Removing LD from the GC + MS configuration decreases PQ from 52.37 to 51.44 and mPQ from 50.80 to 50.31. These results show that the three branches do not simply duplicate one another. GC supplies tree-level context, MS improves semantic discrimination, and LD strengthens fine instance delineation. Their combination obtains the best mIoU, PQ, PQ_T_, and mPQ among the branch-level variants, indicating that the HFE branches are complementary under the same validation protocol.

HFE does not affect all evaluation levels equally (Supplementary Table S4). Relative to Shared Features, the Complete Specialized Design increases mIoU from 61.82 to 65.37, PQ from 47.73 to 52.37, and mPQ from 48.53 to 50.80, corresponding to gains of 3.55, 4.64, and 2.27 points, respectively. PQ_T_, however, decreases slightly from 49.33 to 49.23. The complete design recovers 0.28 points relative to the dilation-only variant (48.95 to 49.23), but remains below the shared-feature baseline on this metric. This result does not support a uniform improvement claim for HFE. Instead, HFE in isolation favors semantic classification and standard-instance delineation at the cost of a small trade-off in tree-level grouping. One possible reason is that task-specific transformations alter the mixture of information retained in the shared encoder representation. The multi-scale semantic and local-detail branches emphasize category context and fine instance boundaries, consistent with the larger gains in mIoU and PQ. The tree branch instead prioritizes long-range structure through dilated convolution and channel recalibration, which may weaken some local cues used to associate spatially sparse fruits and trunks with their parent tree. HFE also has no direct term that enforces the assignment of child components to a parent tree. The combination results in Table 4 help separate these roles. Adding CDAG to HFE raises PQ_T_ from 49.23 to 51.20 through task-adaptive feature selection during decoding; HCL then raises it to 53.37 by constraining parent–child consistency. The full model therefore uses HFE for initial task specialization and relies on CDAG and HCL to address the tree-level weakness observed when HFE is evaluated alone.

#### 4.3.2 Ablation of the CDAG module

The attention components are added progressively in Supplementary Table S6. Spatial attention alone increases mIoU from 61.82 to 64.24 and mPQ from 48.53 to 49.05. Channel attention raises PQ to 51.30 and mPQ to 50.39. Adding multi-scale attention increases PQ_T_ from 49.47 to 49.76 and mPQ to 50.47, although PQ changes slightly from 51.30 to 51.18. The final pixel-level component brings the complete design to 66.74 mIoU, 53.31 PQ, 50.13 PQ_T_, and 51.72 mPQ. The four dimensions work best as a group, but the trajectory is not monotonic for every metric.

This progressive ablation also clarifies the complementarity of the four attention dimensions. Spatial attention alone improves mIoU by 2.42 points and mPQ by 0.52 points over the no-gating baseline, showing that decoder-conditioned location selection is useful. Adding channel attention raises PQ from 49.11 to 51.30 and mPQ from 49.05 to 50.39, indicating that selecting task-relevant feature channels contributes additional information beyond spatial weighting. Adding multi-scale attention increases PQ_T_ from 49.47 to 49.76 and mPQ from 50.39 to 50.47, while PQ changes slightly from 51.30 to 51.18. This metric-specific change suggests that multi-scale attention is not a duplicate of spatial attention; it mainly modifies how local and broader contextual cues are balanced. Finally, pixel-level fusion increases mIoU, PQ, PQ_T_, and mPQ to 66.74, 53.31, 50.13, and 51.72, respectively. The largest additional gain appears in PQ, which is consistent with the role of point-level fusion in refining instance boundaries and mask assignment.

Fig. 5 visualizes the CDAG weights for one representative validation sample. The four rows correspond to spatial weighting, channel weighting, multi-scale weighting, and the final fused pixel-level weighting. These maps are intended to illustrate how the gate modulates skip features for different decoders; they should be interpreted together with the ablation results rather than as standalone causal evidence.

**Fig. 5:**
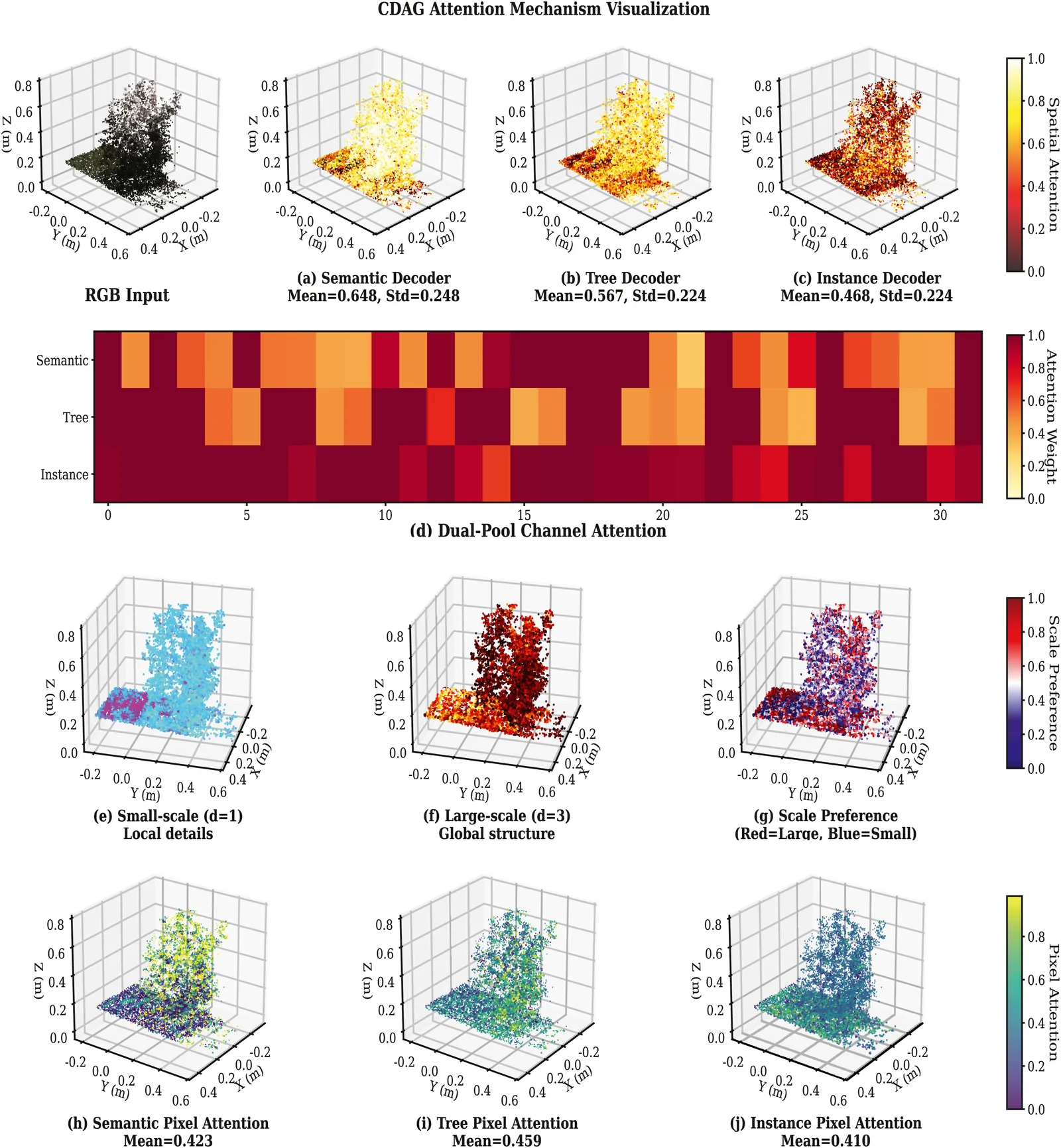
Visualization of four-dimensional attention weights in CDAG.

The first row compares the RGB point cloud with the spatial weights learned for the Semantic, Tree, and Instance Decoders. The three maps differ in magnitude and spatial distribution. In this sample, the semantic map has the highest mean weight, the tree-instance map has the lowest standard deviation, and the standard-instance map has the lowest mean but the highest standard deviation. This pattern is consistent with the different requirements of the three tasks: semantic segmentation benefits from broader contextual activation, tree-level grouping favors smoother responses across the parent structure, and standard-instance segmentation requires more selective emphasis around fine components. Because the visualization is sample-specific, it is not used to assign a universal role to any fixed spatial region.

The second row shows the channel-attention heatmap. The rows correspond to the three decoders, and the horizontal axis indexes the *C* latent feature channels. Several channels receive different weights across tasks, indicating that each decoder selects a different mixture of encoder channels. The channel indices are latent dimensions, so the heatmap is not interpreted as assigning explicit object semantics to individual channels.

The third row shows the responses of the small-dilation branch (*d* = 1), the large-dilation branch (*d* = 3), and the learned preference between them. The preference map contains regions assigned to both receptive-field scales, suggesting that CDAG does not apply a single scale uniformly to the whole point cloud. Instead, it adjusts the balance between local and broader contextual features at different positions. This interpretation is kept at the feature level, because the visualization does not by itself identify which object category is represented by either branch.

The final row presents the fused pixel-level weights supplied to the three decoders after spatial, channel, and multi-scale information has been combined. The distributions remain task-dependent even though all three maps originate from the same encoder representation. For the Instance Decoder, the fused map provides non-uniform point-level emphasis rather than transferring the skip feature uniformly. This behavior is consistent with the role of instance prediction, where local fruit and trunk boundaries require stronger feature selection. The qualitative observation is also consistent with the quantitative ablation: adding pixel-level fusion increases PQ from 51.18 to 53.31 and mPQ from 50.47 to 51.72. Thus, the visualization supports the interpretation that CDAG supplies decoder-specific feature modulation, while the validation-set ablation provides the quantitative evidence for its contribution.

CDAG behaves differently depending on where it is applied (Supplementary Table S7). Restricting it to the Tree Decoder increases mIoU from 61.82 to 64.42, PQ from 47.73 to 51.47, and mPQ from 48.53 to 50.27, but lowers PQ_T_ from 49.33 to 49.06. At this position, the Semantic Decoder supplies the spatial gating signal. Semantic features emphasize category-specific regions and local boundaries, whereas tree-level segmentation must associate spatially separated fruits and trunks. Used alone, this gate may favor locally discriminative information over some cues needed for tree grouping, which is consistent with the gains in mIoU and standard PQ alongside the 0.27-point PQ_T_ decrease. At the Instance Decoder, CDAG instead receives its gating signal from Tree Decoder features, so parent-level structure guides feature selection for fine-grained instances. Under shared-encoder joint optimization, this coupling yields the highest position-specific PQ_T_ of 50.21. The all-decoder setting changes the balance among all three tasks. Compared with the Instance-Decoder-only setting, it raises mIoU from 63.97 to 66.74, PQ from 51.59 to 53.31, and mPQ from 50.90 to 51.72; PQ_T_ changes from 50.21 to 50.13. Relative to no gating, full deployment improves mIoU by 4.92 points, PQ by 5.58 points, PQ_T_ by 0.80 points, and mPQ by 3.19 points. We therefore select the all-decoder setting as the best overall multi-task configuration rather than the setting that maximizes PQ_T_ alone. Because repeated-run statistics are unavailable for this CDAG-position ablation, the 0.08-point difference is reported as an observed metric-specific trade-off and is not attributed to statistical variation.

#### 4.3.3 Ablation of the HCL loss

Varying the HCL weight *λ* reveals a clear optimum among the tested settings (Supplementary Table S8). All four metrics improve when *λ* increases from 0 to 0.05. At *λ* = 0.1, mIoU, PQ, PQ_T_, and mPQ reach 65.76, 51.03, 50.01, and 50.52, respectively, the highest values in this ablation. Raising the weight to 0.2 reduces mPQ to 50.13; at *λ* = 0.5, it falls to 49.37. The other metrics follow the same general trend. A moderate consistency term supports hierarchical learning, but a larger weight competes with the semantic and instance objectives. The complete model therefore uses *λ* = 0.1.

This sensitivity analysis also defines the practical role of HCL. The improvement from *λ* = 0 to *λ* = 0.1 shows that center-field consistency is useful as a regularizer for the annotated tree hierarchy. The degradation at *λ* = 0.2 and *λ* = 0.5 indicates that the mean-alignment term should not dominate the semantic, standard-instance, and tree-instance objectives. This supports treating HCL as a moderate soft constraint rather than as a hard geometric assumption about uniform fruit distribution.

### 4.4 Visualization analysis

Representative predictions from the SfM, TLS, UAV, and UGV test subsets are displayed in Fig. 6. They permit visual inspection of semantic regions and instance assignments under different viewpoints, point densities, and reconstruction characteristics. In these examples, the outputs separate major scene regions and assign distinct identities to detected tree components. Test-set ground truth is unavailable, however, so the correctness of individual labels, the extent of missed or incorrectly segmented regions, and relative performance across sensor domains cannot be determined. These predictions illustrate model behavior on the released point clouds but do not provide quantitative evidence of cross-sensor generalization.

**Fig. 6:**
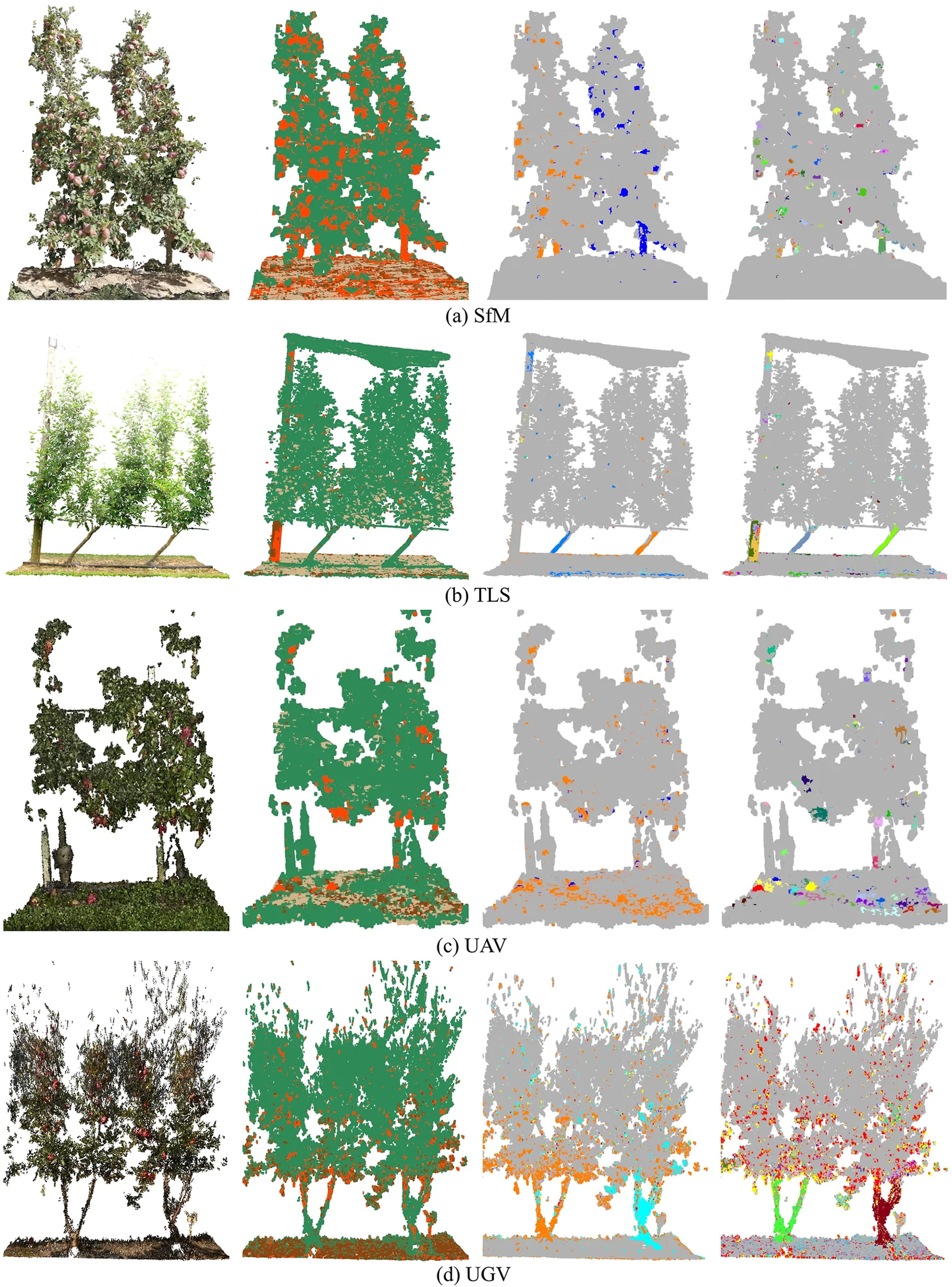
Qualitative HAE-Net predictions on the four HOPS test subsets.

### 4.5 Limitations of the method

Because ground-truth annotations are unavailable for the four test subsets, the quantitative conclusions of this study are limited to the labeled TLS validation split, and the test-set predictions should be interpreted only as qualitative examples. A complete quantitative assessment of cross-sensor performance will require an additional annotated multi-sensor benchmark or access to the withheld test annotations.

Dense fruit clusters and overlapping canopies remain difficult to segment. Under occlusion, standard instances may be missed or merged, and components can be assigned ambiguously to adjacent parent trees. The evaluation scope is also narrow: all labeled training and validation data come from one orchard and one acquisition modality. The current results therefore do not establish transfer to other orchards, crop structures, seasons, or sensors. Evaluation on labeled multi-orchard and multi-sensor data is needed, and temporal observations may provide an additional cue for separating occluded components.

More specifically, HAE-Net may exhibit different failure modes under several challenging orchard conditions. Under severe fruit occlusion, only a small part of a fruit surface may be visible in the point cloud. The Semantic Decoder may then suppress the partially observed fruit as background or canopy, and the Instance Decoder may merge it with nearby organs because the geometric evidence for a separate object is incomplete. In high-density fruit clusters, adjacent fruits often have similar color and local geometry and may be separated by only narrow or missing point gaps. This can lead to under-segmentation, where several neighboring fruits are predicted as one standard instance, or to fragmented boundaries when the visible separation between fruits is inconsistent across viewpoints. Overlapping canopies introduce a different error source: even if the fine fruit instances are detected, the Tree Decoder may assign them to the wrong parent tree when the fruit cluster lies near the interface between adjacent crowns.

Additional failure modes may occur when the test orchard differs from the HOPS distribution. Different cultivars or maturity stages can change fruit size, fruit color, canopy density, and the visibility of trunks and branches. These changes may shift both semantic appearance cues and instance-scale cues, reducing the reliability of features learned from a single orchard. Tree age and tree architecture can also affect performance. Young trees with sparse canopies, mature trees with large irregular crowns, and strongly pruned trees have different canopy scales and different spatial relationships between fruits, trunks, and the parent tree. Such structural variation may weaken the matching between the learned multi-scale features and the true object scale, and it may also reduce the suitability of a mean-based parent–child center constraint when visible child components are strongly asymmetric. These cases indicate that future work should combine occlusion-aware instance separation, cross-cultivar and cross-maturity adaptation, multi-orchard validation, and more flexible hierarchical constraints for diverse tree ages and architectures.

Another limitation concerns the mean-based form of HCL. Although the loss is applied only as a soft training regularizer, the arithmetic mean can be affected by strongly skewed fruit visibility, partial scans, or severe occlusion. A robust extension would replace the unweighted mean with a confidence- or density-weighted center, so that unreliable or sparsely observed components contribute less to the parent–child consistency term. Another possible extension is to use a median-based or geometric-median center estimator, which would reduce the influence of outlying predicted centers. These alternatives were not evaluated in the present study and should be tested in future work before being used as replacements for the current HCL formulation.

A further limitation is that the quantitative evaluation is currently limited to the HOPS dataset. This limitation reflects the early stage of hierarchical tree-level panoptic segmentation in agricultural and forestry point clouds, where benchmark construction remains substantially more difficult than ordinary semantic or single-level instance annotation. The full HAE-Net evaluation, especially HCL, requires parent–child panoptic annotations linking fine fruit or trunk instances to tree-level instances. Recent reviews of 3D plant phenomics also identify benchmark construction, efficient point-cloud analysis, and model extensibility as continuing challenges [49]. At the same time, the emergence of tree-structured panoptic point-cloud benchmarks suggests that this problem setting is becoming a broader research direction rather than an isolated dataset-specific task [50]. Future work should evaluate HAE-Net on multi-orchard, multi-crop, and multi-sensor point-cloud benchmarks with explicit parent–child instance annotations, and should examine whether HFE and CDAG transfer to general organ-level segmentation while HCL is adapted to other biological hierarchies.

## 5 Conclusion

This paper presents HAE-Net for hierarchical 3D panoptic segmentation in orchard scenes. Its three components act at different points in the network. HFE transforms the shared encoder representation through task-oriented branches: global-context aggregation and channel recalibration support tree-level grouping, multi-scale fusion balances semantic context and boundaries, and local-detail enhancement supports standard-instance delineation. During decoding, CDAG uses spatial, channel, multi-scale, and pixel-level attention to regulate the skip features supplied to each task. HCL acts on the training objective by aligning the centers predicted for child components with those of their parent tree instances. This design introduces task specialization without removing information exchange across the hierarchy. On the labeled TLS validation split, HAE-Net outperforms the compared methods in semantic segmentation (mIoU), panoptic segmentation (PQ), tree-level panoptic segmentation (PQ_T_), and their hierarchical aggregate (mPQ), while the ablation studies quantify the contributions of the proposed modules. Predictions on the four test subsets provide qualitative examples under different acquisition settings but do not establish quantitative cross-sensor performance.

## Author contributions

S.Z., Z.J., and W.J. conceived the study. S.Z. and X.C. curated the data. S.Z., L.S., and Y.W. developed the code and designed and conducted the experiments. S.Z., Y.W., L.S., and X.C. contributed to visualization and validation. S.Z., Z.J., and W.J. prepared the original draft.

## Data availability

The HOPS dataset analyzed in this study is publicly available at https://www.ipb.uni-bonn.de/data/hops/index.html. The trained HAE-Net model weights and codes for running inference on HOPS point clouds are publicly available at https://github.com/PlantPhenotyping/HAE-Net.

## Funding

This work was supported by the 10.13039/501100001809National Natural Science Foundation of China (No. U24A20231, 62372278), the Jinan City–University Integration Development Project (JNSX2025008 and 202534102), Shandong Provincial Natural Science Foundation (No. ZR2025ZD14), and the Taishan Scholar Program of Shandong Province, China.

## Declaration of competing interest

The authors declare that they have no known competing financial interests or personal relationships that could have appeared to influence the work reported in this paper.

## References

[1] Benti N.E., Chaka M.D., Semie A.G., Warkineh B., Soromessa T. (2024). Transforming agriculture with machine learning, deep learning, and IoT: perspectives from Ethiopia–challenges and opportunities. Discover Agricult..

[2] Jabed M.A., Murad M.A.A. (2024). Crop yield prediction in agriculture: a comprehensive review of machine learning and deep learning approaches, with insights for future research and sustainability. Heliyon.

[3] Sa I., Ge Z., Dayoub F., Upcroft B., Perez T., McCool C. (2016). DeepFruits: a fruit detection system using deep neural networks. Sensors.

[4] Bargoti S., Underwood J. (2017). 2017 IEEE International Conference on Robotics and Automation (ICRA).

[5] Kamilaris A., Prenafeta-Boldú F.X. (2018). Deep learning in agriculture: a survey. Comput. Electron. Agric..

[6] Gené-Mola J., Sanz-Cortiella R., Rosell-Polo J.R., Morros J.-R., Ruiz-Hidalgo J., Vilaplana V., Gregorio E. (2020). Fruit detection and 3D location using instance segmentation neural networks and structure-from-motion photogrammetry. Comput. Electron. Agric..

[7] Marks E., Magistri F., Stachniss C. (2022). 2022 IEEE International Conference on Robotics and Automation (ICRA).

[8] Sodano M., Magistri F., Marks E., Hosn F., Zurbayev A., Marcuzzi R., Malladi M.V.R., Behley J., Stachniss C. (2025). 2025 IEEE/RSJ International Conference on Intelligent Robots and Systems (IROS).

[9] Du R., Li S., Xu K., Cheng L., Jiang Y. (2026). Dynamic sparse point voxel transformer for 3D point cloud instance segmentation of dormant apple trees. Plant Phenomics.

[10] Zhang J., Gu J., Hu T., Wang B., Xia Z. (2024). An image segmentation and point cloud registration combined scheme for sensing of obscured tree branches. Comput. Electron. Agric..

[11] Hu Q., Yang B., Xie L., Rosa S., Guo Y., Wang Z., Trigoni N., Markham A. (2020). Proceedings of the IEEE/CVF Conference on Computer Vision and Pattern Recognition.

[12] Yan J., Tan F., Li C., Jin S., Zhang C., Gao P., Xu W. (2024). Stem–leaf segmentation and phenotypic trait extraction of individual plant using a precise and efficient point cloud segmentation network. Comput. Electron. Agric..

[13] Choy C., Gwak J., Savarese S. (2019). Proceedings of the IEEE/CVF Conference on Computer Vision and Pattern Recognition.

[14] Halstead M., Ahmadi A., Smitt C., Schmittmann O., McCool C. (2021). Crop agnostic monitoring driven by deep learning. Front. Plant Sci..

[15] Zhu X., Cheng M., Wu S., Cheng Z., Liu L., Li Y., Guo X. (2024). Crops3D: a 3D dataset of organ-level panoptic segmentation and high-precision phenotyping for maize and sorghum. Sci. Data.

[16] Gené-Mola J., Sanz-Cortiella R., Rosell-Polo J.R., Morros J.-R., Ruiz-Hidalgo J., Vilaplana V., Gregorio E. (2020). Fuji-SfM dataset: a collection of annotated images and point clouds for fuji apple detection and location using structure-from-motion photogrammetry. Data Brief.

[17] Kirillov A., He K., Girshick R., Rother C., Dollár P. (2019). Proceedings of the IEEE/CVF Conference on Computer Vision and Pattern Recognition.

[18] Zhou Z., Zhang Y., Foroosh H. (2021). Proceedings of the IEEE/CVF Conference on Computer Vision and Pattern Recognition.

[19] Narita G., Seno T., Ishikawa T., Kaji Y. (2019). 2019 IEEE/RSJ International Conference on Intelligent Robots and Systems (IROS).

[20] McInnes L., Healy J., Astels S. (2017). HDBSCAN: hierarchical density based clustering. J. Open Source Softw..

[21] Comaniciu D., Meer P. (2002). Mean shift: a robust approach toward feature space analysis. IEEE Trans. Pattern Anal. Mach. Intell..

[22] Gasperini S., Mahani M.-A.N., Marcos-Ramiro A., Navab N., Tombari F. (2021). Panoster: End-to-end panoptic segmentation of LiDAR point clouds. IEEE Rob. Autom. Lett..

[23] Sirohi K., Mohan R., Büscher D., Burgard W., Valada A. (2022). EfficientLPS: Efficient LiDAR panoptic segmentation. IEEE Trans. Robot..

[24] Yang B., Wang J., Clark R., Hu Q., Wang S., Markham A., Trigoni N. (2019). https://proceedings.neurips.cc/paper/2019/hash/86c87222b4e835ee79d4ac8b7ceb4be2-Abstract.html.

[25] Schult J., Engelmann F., Hermans A., Litany O., Tang S., Leibe B. (2023). 2023 IEEE International Conference on Robotics and Automation (ICRA).

[26] Pan Y., Cui Q., Yang X., Zhao N. (2025). Proceedings of the 42nd International Conference on Machine Learning, Vol. 267 of Proceedings of Machine Learning Research.

[27] Li X., Li H., Kong X., Ji Y., Liu Z., Liu H. (2026). Panoptic-VSNet: Visual-semantic prior knowledge-driven multimodal 3D panoptic segmentation. Pattern Recogn..

[28] Song H., Wen W., Wu S., Guo X. (2025). Comprehensive review on 3D point cloud segmentation in plants. Artif. Intell. Agricult..

[29] Dong S., Fan X., Li X., Liang Y., Zhang M., Yao W., Yang X., Wang Z. (2025). Automatic 3D plant organ instance segmentation method based on PointNeXt and Quickshift++. Plant Phenomics.

[30] Jiang L., Li C., Fu L. (2025). Apple tree architectural trait phenotyping with organ-level instance segmentation from point cloud. Comput. Electron. Agric..

[31] Liu T., Sun W., Jiang H., Tian L., Fu X., Jin C., Wang C., Chen C., Hu F. (2026). A holistic 3D framework for single-plant point cloud segmentation and phenotypic trait extraction. Comput. Electron. Agric..

[32] Ruder S. (2017). An overview of multi-task learning in deep neural networks. arXiv preprint arXiv:1706.05098.

[33] Eigen D., Fergus R. (2015). Proceedings of the IEEE International Conference on Computer Vision.

[34] Chen L.-C., Barron J.T., Papandreou G., Murphy K., Yuille A.L. (2016). Proceedings of the IEEE Conference on Computer Vision and Pattern Recognition.

[35] Amrani A., Diepeveen D., Murray D., Jones M.G.K., Sohel F. (2024). Multi-task learning model for agricultural pest detection from crop-plant imagery: a bayesian approach. Comput. Electron. Agric..

[36] Chao X., Yang S. (2026). A generative AI-enhanced multitask learning framework for weed detection and drivable area segmentation in smart agriculture. Comput. Electron. Agric..

[37] Kendall A., Gal Y., Cipolla R. (2018). Proceedings of the IEEE Conference on Computer Vision and Pattern Recognition.

[38] Chen Z., Badrinarayanan V., Lee C.-Y., Rabinovich A. (2018). Proceedings of the 35th International Conference on Machine Learning, Vol. 80 of Proceedings of Machine Learning Research.

[39] Oktay O., Schlemper J., Folgoc L.L., Lee M., Heinrich M., Misawa K., Mori K., McDonagh S., Hammerla N.Y., Kainz B., Glocker B., Rueckert D. (2018). Attention U-Net: learning where to look for the pancreas. arXiv preprint arXiv:1804.03999.

[40] Si Y., Xu H., Zhu X., Zhang W., Dong Y., Chen Y., Li H. (2025). SCSA: exploring the synergistic effects between spatial and channel attention. Neurocomputing.

[41] Chen Z., He Z., Lu Z.-M. (2024). DEA-Net: single image dehazing based on detail-enhanced convolution and content-guided attention. IEEE Trans. Image Process..

[42] Woo S., Park J., Lee J.-Y., Kweon I.S. (2018). Proceedings of the European Conference on Computer Vision.

[43] Huang H., Chen Z., Zou Y., Lu M., Chen C., Song Y., Zhang H., Yan F. (2024). Channel prior convolutional attention for medical image segmentation. Comput. Biol. Med..

[44] Berman M., Triki A.R., Blaschko M.B. (2018). Proceedings of the IEEE Conference on Computer Vision and Pattern Recognition.

[45] Marcuzzi R., Nunes L., Wiesmann L., Behley J., Stachniss C. (2023). Mask-based panoptic LiDAR segmentation for autonomous driving. IEEE Rob. Autom. Lett..

[46] Malladi M.V.R., Chebrolu N., Scacchetti I., Lobefaro L., Guadagnino T., Casseau B., Oh H., Freißmuth L., Karppinen M., Schweier J., Leutenegger S., Behley J., Stachniss C., Fallon M. (2025). 2025 IEEE International Conference on Robotics and Automation (ICRA).

[47] Xiao Z., Zhang W., Wang T., Loy C.C., Lin D., Pang J. (2025). Position-guided point cloud panoptic segmentation transformer. Int. J. Comput. Vis..

[48] Robert D., Raguet H., Landrieu L. (2024). International Conference on 3D Vision (3DV).

[49] Jin S., Li D., Yun T., Tang J., Wang K., Li S., Yang H., Yang S., Xu S., Cao L., Xia H., Guo Q., Zhang Y., Jiang D., Ding Y. (2025). Deep learning for three-dimensional (3d) plant phenomics. Plant Phenomics.

[50] Novoa Comesaña S., Méndez Vales V., Suárez-Fernández G.E., Ruiz Maíz M., Martínez-Sánchez J. (2026). 3D-TREES-PAN: a LiDAR point cloud dataset for forest structure instance-level tree panoptic segmentation. Embarg. Until March.

