# Supplementary material for: HAE-Net: A hierarchy-aware enhanced method for 3D panoptic segmentation in orchard scenes
Source: Plant Phenomics. 2026 Sep 10;8(4):100273. doi: 10.1016/j.plaphe.2026.100273 (PMC13634764; doi:10.1016/j.plaphe.2026.100273)
Supplement: Supplementary file 1 [file mmc1.pdf]

Supplementary Materials for
HAE-Net: A Hierarchy-Aware Enhanced Method for 3D Panoptic Segmentation in Orchard Scenes

Shuo Zhang[a], Yiqun Wang[a], Lianchuan Shi[a], Ze Ji[b], Xiude Chen[c], Weikuan Jia[a,*]

[a] *School of Computer Science and Artificial Intelligence Shandong Normal University Jinan 250358 China*
[b] *School of Engineering Cardiff University Cardiff CF24 3AA United Kingdom*
[c] *National Research Center for Apple Engineering and Technology Shandong Agricultural University Taian 271018 China*

Overview

HAE-Net predicts semantic labels, standard fruit/trunk instances, and parent tree instances from orchard point clouds. This document provides the supporting information needed to interpret the supplementary tables without relying on table entries alone. Supplementary Methods S1–S5 describe the HOPS data splits and label hierarchy, the common experimental settings and evaluation metrics, and the controlled designs of the HFE, CDAG, and HCL ablations. Tables S1–S8 then report the corresponding dataset statistics, configurations, and detailed results.

Performance metrics in Tables S4–S8 are reported on a 0–100 scale and are evaluated on the labeled HOPS TLS validation split. A dash in a table denotes information that cannot be calculated from the publicly released annotations; it does not represent a measured value of zero.

Contents

Supplementary Methods
S1. Dataset composition and label hierarchy
S2. Experimental settings and evaluation metrics
S3. Controlled design of the HFE ablations
S4. Controlled design of the CDAG ablations
S5. Controlled design of the HCL loss-weight analysis

Supplementary Tables
Table S1. Statistics of the HOPS dataset
Table S2. Semantic category definitions in the HOPS dataset
Table S3. Experimental configurations
Table S4. Ablation of the HFE module
Table S5. Branch-level ablation of the HFE module
Table S6. Ablation of multi-dimensional attention in CDAG
Table S7. Ablation of CDAG application positions
Table S8. Sensitivity analysis of the HCL loss weight λ

*Corresponding author.

Supplementary Methods

S1. Dataset composition and label hierarchy

The HOPS release used in this study contains labeled terrestrial-laser-scanner (TLS) training and validation splits, together with TLS, uncrewed-aerial-vehicle (UAV), uncrewed-ground-vehicle (UGV), and Structure-from-Motion (SfM) test subsets. Table S1 reports the number of samples and average point count for each split. The public training and validation data include semantic, standard-instance, and tree-instance annotations. The public test subsets contain point clouds without these ground-truth labels; consequently, their average fruit and trunk counts cannot be calculated and no quantitative test-subset metrics are reported.

The semantic taxonomy is summarized in Table S2. The void label is ignored during evaluation. Ground, plant/canopy, and pole are "stuff" classes, whereas fruit and trunk are "things" classes. At the fine-grained level, every fruit and trunk has a standard-instance identity. At the parent level, one tree instance comprises one trunk and all fruits assigned the same tree identity. Thus, points on components of the same physical tree share a tree-instance label while retaining their separate standard-instance labels.

S2. Experimental settings and evaluation metrics

Table S3 lists the network, optimization, data-augmentation, early-stopping, and computing settings used for the experiments. The detailed single-run comparisons and ablation studies use random seed 42. Unless a module is explicitly enabled in an ablation, the remaining architecture and training settings are held fixed so that the reported change can be attributed to the tested configuration.

Four metrics are used. Mean intersection over union (mIoU) measures semantic segmentation across the evaluated classes. Standard panoptic quality (PQ) treats individual fruits and trunks as separate instances. Tree-level panoptic quality (PQ_T) instead treats one trunk and its associated fruits as a parent tree instance. Mean panoptic quality (mPQ) is the arithmetic mean of PQ and PQ_T. An upward arrow in a table indicates that a higher value is better.

S3. Controlled design of the HFE ablations

Tables S4 and S5 evaluate the Hierarchical Feature Enhancement (HFE) module while the Cross-Decoder Attention Gate (CDAG) and Hierarchical Consistency Loss (HCL) are disabled. Table S4 first compares three encoder-output designs. "Shared Features (No HFE)" sends the same encoder representation to all three decoders. "Only Dilation Differences" retains decoder-specific convolutional branches with dilation rates $d = 1, 2, 4$ but omits the task-oriented enhancement operations. "Complete Specialized Design" adds global-context aggregation and channel recalibration for the Tree Decoder, multi-scale semantic fusion for the Semantic Decoder, and local-detail enhancement for the Instance Decoder.

Table S5 uses the dilation-only design as the controlled reference and enables the three specialized branches individually and in combination. GC denotes the global-context tree branch, MS the multi-scale semantic branch, and LD the local-detail instance branch. A check mark indicates that the corresponding enhancement is enabled, and a cross indicates that it is disabled. This design separates the metric-specific contribution of each branch from the effect of merely using different dilation rates.

S4. Controlled design of the CDAG ablations

Tables S6 and S7 evaluate CDAG while HFE and HCL are disabled. Table S6 uses nested configurations to add spatial, channel, multi-scale, and pixel-level attention progressively. Each row therefore retains the dimensions listed in the preceding row and adds one new attention operation. Table S7 holds the complete four-dimensional gate fixed and varies the decoder positions at which it is applied. A check mark denotes a decoder that uses CDAG, and a cross denotes direct feature transfer without CDAG. The position study reports the task-specific trade-off between the setting applied only to the Instance Decoder and the setting applied to all three decoders, while mPQ summarizes performance across the two instance granularities.

S5. Controlled design of the HCL loss-weight analysis

Table S8 evaluates the Hierarchical Consistency Loss (HCL) while HFE and CDAG are disabled. The weight λ controls the contribution of HCL to the joint training objective; $\lambda = 0$ is the no-HCL reference. The values 0.05, 0.1, 0.2, and 0.5 test whether the center-field consistency term is most effective as a moderate regularizer or as a dominant constraint. The selected value, $\lambda = 0.1$, gives the highest mIoU, PQ, PQ_T, and mPQ among the tested settings and is used in the complete HAE-Net configuration.

Supplementary Tables

Table S1: Statistics of the HOPS dataset. Fruit and trunk counts are unavailable for the test subsets because their ground-truth instance annotations are not included in the public release.

Metric	Training Set (TLS)	Validation Set (TLS)	Test Set UGV	Test Set UAV	Test Set SfM	Test Set TLS
Number of samples	90	18	12	22	6	27
Average points	0.8M	1.0M	0.6M	1.5M	1.8M	1.0M
Average fruits	90.2	99.2	–	–	–	–
Average trunks	3.1	3.2	–	–	–	–

Table S2: Semantic category definitions in the HOPS dataset.

Class ID	Class Name	Type	Description
0	void	–	Ignored
1	ground	Stuff	Ground
2	plant	Stuff	Plant/Canopy
3	fruit	Things	Fruit
4	trunk	Things	Trunk
5	pole	Stuff	Pole

Table S3: Experimental configurations used for the single-run comparisons and ablation studies.

Configuration Item	Parameter Value	Configuration Item	Parameter Value
**Basic Network Config**		**Optimizer Config**	
Backbone	MinkUNet	Optimizer Type	AdamW
Voxel Resolution	3 mm	Initial Learning Rate	5×10^{-3}
Standard Instance Variance	0.01	Minimum Learning Rate	1×10^{-6}
Tree Instance Variance	0.5	Weight Decay	0.01
Baseline Method	HAPT3D	Adam beta parameters	[0.9, 0.999]
**Early Stopping Strategy**		**Learning Rate Scheduling**	
Monitor Metric	mPQ (Validation Set)	Scheduling Strategy	Cosine Annealing
Patience	50 epochs	Warmup Epochs	10 epochs
Warmup Period	100 epochs	Maximum Training Epochs	500 epochs
**Data Augmentation**		**Training Config**	
Random Scaling	0.8–1.2×	Batch Size	1
Random Rotation (X/Y axes)	±15°	Random Seed	42
Random Rotation (Z axis)	±180°	**Hardware and Software**	
Random Shear Coefficient	0.2	CPU	Intel Xeon Gold 6226R
Random Subsampling Ratio	0.6–1.0	GPU	NVIDIA A30
Color Contrast	0.8–1.2	Operating System	Ubuntu 18.04
Color Brightness Jitter	±0.2	CUDA	11.3
Hue Jitter	±0.15	PyTorch	1.12.1
Saturation Jitter	±0.15	Python	3.9.29

Table S4: Ablation of the HFE module with CDAG and HCL disabled.

HFE Design	mIoU	PQ	PQ_T	mPQ
Shared Features (No HFE)	61.82	47.73	49.33	48.53
Only Dilation Differences ($d = 1, 2, 4$)	64.88	51.44	48.95	50.20
Complete Specialized Design (Ours)	65.37	52.37	49.23	50.80

Table S5: Branch-level ablation of the HFE module with CDAG and HCL disabled. The dilation-only design is the controlled reference.

GC	MS	LD	mIoU↑	PQ↑	PQ_T↑	mPQ↑
✗	✗	✗	64.88	51.44	48.95	50.20
✓	✗	✗	64.91	51.46	49.18	50.32
✗	✓	✗	65.24	51.43	48.99	50.21
✗	✗	✓	64.98	52.21	48.95	50.58
✓	✓	✗	65.27	51.44	49.21	50.31
✓	✗	✓	64.99	52.22	49.20	50.71
✗	✓	✓	65.31	52.31	48.96	50.64
✓	✓	✓	65.37	52.37	49.23	50.80

Table S6: Ablation of multi-dimensional attention in CDAG with HFE and HCL disabled.

Attention Dimensions	mIoU	PQ	PQ_T	mPQ
No gating	61.82	47.73	49.33	48.53
Spatial only	64.24	49.11	49.06	49.05
Spatial + Channel	64.74	51.30	49.47	50.39
Spatial + Channel + Multi-scale	65.13	51.18	49.76	50.47
Spatial + Channel + Multi-scale + Pixel	66.74	53.31	50.13	51.72

Table S7: Ablation of CDAG application positions with HFE and HCL disabled.

Semantic Decoder	Tree Decoder	Instance Decoder	mIoU	PQ	PQ_T	mPQ
✗	✗	✗	61.82	47.73	49.33	48.53
✓	✗	✗	63.78	49.22	49.30	49.26
✗	✓	✗	64.42	51.47	49.06	50.27
✗	✗	✓	63.97	51.59	50.21	50.90
✓	✓	✓	66.74	53.31	50.13	51.72

Table S8: Sensitivity analysis of the HCL loss weight λ with HFE and CDAG disabled.

λ	mIoU	PQ	PQ_T	mPQ
0	61.82	47.73	49.33	48.53
0.05	64.57	50.73	49.48	50.11
0.1	65.76	51.03	50.01	50.52
0.2	64.22	50.77	49.48	50.13
0.5	62.11	49.56	49.17	49.37
